# *In vitro* characterization of *drynaria in-situ* gel: A novel approach to periodontal disease management

**DOI:** 10.6026/973206300212006

**Published:** 2025-07-31

**Authors:** Kaushal Pati Tripathi, Neelam Mittal, Anju Gautam, Pragati Dubey, Aishwarya Pandey, Sakshi Gupta, Ajay Kumar, Varsha Choubey

**Affiliations:** 1 Department of Periodontology, Institute of Medical Sciences, Banaras Hindu University, Varanasi-221005, Uttar Pradesh, India; 2 Department of Conservative Dentistry and Endodontics, Faculty of Dental Sciences, Institute of Medical Sciences, Banaras Hindu University, Varanasi-221005, Uttar Pradesh, India; 3 Institute of Medical Sciences, Banaras Hindu University, Varanasi-221005, Uttar Pradesh, India; 4 Unit of Oral Medicine and Radiology, Institute of Medical Sciences, Banaras Hindu University, Varanasi-221005, Uttar Pradesh, India; 5 Department of Periodontics and Implantology, Hitkarini Dental College and Hospital, Jabalpur-482001, India

**Keywords:** *drynaria in-situ* gel, evaluation, periodontal diseases

## Abstract

The condition of periodontal diseases that results from oral dysbiosis-related inflammation poses crucial public health concerns. Therefore, it is of interest to assess an in-situ gel made with *Drynaria rhizome* extract for local drug application in periodontal care. The *drynaria in-situ* gel has desirable clinical properties because its combination of Sodium Tripolyphosphate (STPP), Poloxamer 407, Acetic Acid and Chitosan produces temperature-sensitive features and viscosity stability. Studies under laboratory conditions proved both the uniformity and stability of the gel. The tested *Drynaria gel* emerges as a promising choice for local drug delivery in periodontal healthcare and researchers need to conduct additional clinical trials to prove its performance in real-world conditions.

## Background:

Periodontal diseases affect the teeth's supporting structures by causing inflammation which damages the gingiva together with the periodontal ligament and alveolar bone along with cementum. These diseases compose a main global health problem because advanced cases often result in lost teeth and systemic medical problems [1]. Periodontitis exists as a persistent medical condition that healthcare professionals classify number six worldwide with over 743 million persons affected [2]. The condition creates damage to someone's oral fitness alongside destruction of personal confidence along with injuries to their whole body system and their overall quality of life. Periodontal disease starts when inflamed conditions appear in the gingival tissue named gingivitis. Periodontal bacteria that include *Porphyromonas gingivalis* (*Pg.*) *Aggregatibacter actinomycetemcomitans* (*Aa.*), *Prevotella intermedia* (*Pi.*) and *Fusobacterium nucleatum* (*Fn.*) among more than fifteen others can proliferate when pockets develop due to attachment loss and alveolar bone damage along with periodontal ligament and cementum involvement [3, 4]. The essential practice for periodontal infection control includes both scaling and root planning (SRP) procedures and disciplined home oral hygiene practices. The removal of pathogenic bacteria which adopt a biofilm structure requires these procedures because biofilms are the primary microbial cause of periodontitis [5]. Local biofilm reduction through mechanical treatments should be supplemented with host-modulating agents who enhance periodontal treatment outcomes. The achievement of best therapeutic effects with reduced systemic therapy-related risks depends on vital controlled molecule delivery systems. Judging by current evidence both SRP and a local drug delivery system should work together for optimal therapeutic results according to studies in [6, 7]. Local drug delivery (LDD) methods have emerged as critical formulations that focus therapeutic drugs directly on specific sites throughout the periodontal area. Therapy effectiveness depends heavily on how medication release occurs together with the chosen dosage form delivery methods. Systemic antimicrobial medications work effectively if the administered doses reach proper levels inside periodontal pockets according to recommendations [7]. The medical benefits from antimicrobial agent usage through systemic routes are canceled out by the increasing microbial drug resistance and adverse effects. Direct delivery of therapeutic medications through LDD systems achieves maximal treatment outcomes while minimizing side effects because the devices directly inject treatments into periodontal pockets 8. Periodontal pockets work as natural storage areas to host LDD placements. The periodontal pockets' liquid substance known as GCF supports drug dispersion through time which leads to improved drug effectiveness [8].

Ayurvedic indigenous medicines have gained new significance because researchers pursue alternative and nature-based treatment options. *Drynaria quercifolia* represents a potent medicinal plant studied as a promising agent for oral health biology together with regenerative dentistry because it originates from many Asian territories including India [9]. Researchers explain Gol-Se-Bo as the traditional Korean name for this herb which has Chinese identification as Gu-Sui-Bu and holds the scientific title Aglaomorpha in India. The traditional Ayurvedic medicine has employed *Drynaria quercifolia* plant as a standard therapy for its content of flavonoids, phenolic acids, triterpenoids and other bioactive compounds. It was initially used to address inflammation, hyperlipidemia, rheumatism, osteoporosis and oral bacterial infections [10, 11]. This herb has shown antimicrobial, anti-inflammatory and bone regenerative properties, making it a suitable herbal alternative for the development of LDD systems in the management of periodontal diseases [10, 11 and 12]. The extract derived from the *Drynaria rhizome*, containing flavonoid and triterpenoid admixtures, has been demonstrated to enhance the levels of intracellular total proteins and alkaline phosphatase, as well as improve bone cell viability [11]. The studies presented by researchers showed how *Drynaria quercifolia* rhizome proved effective as a periodontal solution by improving human gingival fibroblast attachment and growth during laboratory tests [13]. Therefore, it is of interest to create and evaluate *Drynaria quercifolia*-based gel because this new agent could serve as a local treatment method for periodontal diseases.

## Materials and Methods:

## Procurement and extraction of plant material:

Fresh Drynaria plants were collected from Udhagamandalam, Tamil Nadu and India. The rhizomes were carefully cut into small sections, shade-dried to preserve phytochemical integrity and then pulverized into a fine powder using a mechanical grinder. The powdered rhizome was stored in an air-tight container until further analysis.

## Extraction process:

A total of 3 kg of the powdered rhizome underwent maceration using ethanol as the solvent. After the extraction period, the mixture was concentrated using a rotary vacuum evaporator, yielding 12 grams (w/w) of dried ethanolic extract. This extract served as the active ingredient for in situ gel formulation (Figure 1 - see PDF).

## Preparation of *drynaria in-situ* gel:

To prepare the gel, sodium tripolyphosphate (STPP) and Poloxamer 407 were dispersed in 50 mL of distilled water under cold conditions, allowing for a two-hour swelling period. This mixture was stirred continuously with a magnetic stirrer until it transitioned into a gel. Separately, methylparaben was dissolved in distilled water using gentle heat in a water bath. Once cooled to room temperature, this solution was combined with the first and propylene glycol was added to enhance the formulation. The ethanolic extract of *Drynaria rhizome* (20 mg) was incorporated into the mixture and the final volume was adjusted to 100 mL with distilled water. Acetic acid and chitosan were added dropwise to adjust the pH to approximately 6.8-7.0, allowing the gel to form homogeneously (Table 1 - see PDF).

## *In vitro* characterization of the gel:

## Physical appearance and gelation capacity:

The gel was visually inspected for clarity and transparency. To assess gelation, 100 µL of the formulation was added to 2 mL of artificial tear fluid maintained at 35°C. The time required for gel formation was noted through visual observation.

## Viscosity and spread ability:

Viscosity was measured using a Brookfield viscometer (spindle no. 01) at 4°C and 37°C, both under 20 rpm. Each measurement was repeated thrice for accuracy. Spread ability was tested by sandwiching the gel between two standard-diameter glass slides. A 100 g weight was placed on the upper slide to spread the gel into a uniform layer (Figure 2 - see PDF).

## pH evaluation:

The gel's pH was measured by dissolving 1 g of the formulation in 10 mL of filtered water. The pH meter was calibrated with a standard buffer solution (pH 7) prior to each reading. The electrode was immersed in the sample for ten minutes at room temperature and the pH readings were recorded in triplicate.

## Drug content uniformity:

To assess drug content, 1 g of the formulation was dissolved in 5 mL of methanol in a 50 mL volumetric flask. The solution was filtered through Whatman filter paper and 0.1 mL of the filtrate was diluted to 10 mL with methanol. The drug concentration was analyzed using a UV-Vis spectrophotometer at 280 nm, using a reference calibration curve. Additionally, FTIR spectroscopy was employed to identify functional groups in the Drynaria extract and to examine any potential chemical interactions between the extract and formulation excipients, such as chitosan, Poloxamer 407, STPP and acetic acid.

## Syringes pass ability:

Following the method described by Maheshwari *et al.* (2006), the syringe-ability of the in-situ gel was tested by evaluating its ability to pass through a 21-gauge needle. One milliliter of the chilled gel was loaded into the syringe and its flow was assessed under standard handling pressure.

## Morphological and thermal characterization:

High-power optical microscopy was used to observe the structural features of the Drynaria extract. Scanning Electron Microscopy (SEM) was employed to examine the surface morphology of the in-situ gel, revealing its textural properties and identifying any particulate matter or aggregates. Differential Scanning Calorimetry (DSC) was also conducted to investigate the thermal properties of the extract and its final gel formulation, enabling the identification of thermal transitions during the formulation process.

## *In vitro* drug release studies:

The *In vitro* drug release of the *Drynaria gel* was evaluated using dialysis membrane bags (Visking, 12-14 kD cut-off; SERVA, Germany). One milliliter of 1% gel or 0.5 mL of 2% gel (containing 10 mg of Drynaria) was enclosed in dialysis bags and immersed in 50 mL of ethanol:water (1:1) at 37 ± 0.5°C. The assembly was maintained in a shaking water bath at 25 rpm. At predetermined intervals, 1 mL aliquots were withdrawn and replaced with fresh medium. Drug release was quantified spectrophotometrically at 650 nm, using a curcumin calibration curve prepared in ethanol: water (1:1). The entire release study was conducted over a period of 7 days, with daily sampling and each experiment was repeated thrice as per the methodology by Dias *et al.* (2019) [30].

## *In vitro* antibacterial activity:

The antibacterial efficacy of the *drynaria in-situ* gel was assessed via the disc diffusion method, following the protocol described by Zhang *et al.* (2016) [31]. The study was conducted against *Staphylococcus aureus* strain 902, obtained from MTCC, Chandigarh, India. Muller Hinton Agar (MHA) plates containing 15 mL of media were inoculated with bacterial cultures standardized to 10^7^ CFU/mL (0.5 McFarland standard) using sterile saline. Wells were filled with the in-situ gel and the plates were incubated at 37°C for 24 hours. Zones of inhibition were measured using a transparent ruler on days 1, 3, 7 and 14 to evaluate the gel's sustained antibacterial effect.

## Results:

The Drynaria-based in-situ gel formulation exhibited excellent physical characteristics. During the formulation process, the gel formed as a clear and transparent substance (Table 2 - see PDF), which is essential for uniform drug distribution and contributes to both medicinal efficacy and patient compliance. The clarity of the gel also indicates the absence of undissolved particles, supporting the visual acceptability of the preparation. The gelling capacity of the formulation was assessed at body temperature (37°C), where it transformed into a gel within approximately 50 ± 1.2 seconds, indicating its suitability for clinical application (Figure 1 - see PDF). The pH of the *drynaria in-situ* gel was measured to be around 4, which, although slightly acidic, remains within an acceptable range for intraoral use. Upon placement in the periodontal pocket, the formulation encounters the neutral physiological pH and body temperature, facilitating gelation and ensuring good mucoadhesion. Viscosity testing showed a marked increase from 58.7 ± 1.8 cps at 25°C to 245.2 ± 22.6 cps at 37°C, demonstrating its temperature-responsive nature, which aids in site retention post-application (Table 4 - see PDF). Spread ability results confirmed that a 100 g weight was sufficient to produce uniform spreading of the gel between glass slides and the formulation exhibited consistent texture, enhancing its ease of application (Table 3 - see PDF). Spectrophotometric analysis of drug content revealed a uniform distribution of Drynaria extract throughout the gel, with consistent drug concentrations across samples, ensuring dosage accuracy (Table 4 - see PDF).

Fourier Transform Infrared (FTIR) spectroscopy was used to identify chemical interactions between the extract and formulation components. Key peaks in the hydroxyl (3200-3500 cm^-1^) and carbonyl (1700 cm^-1^) regions indicated hydrogen bonding and potential complex formation with chitosan, STPP and Poloxamer 407, which contribute to the gel's structural stability. Optical microscopy provided images that depicted the microstructural characteristics of the formulation. These observations showed heterogeneous but uniformly dispersed plant particles, indicating even extract distribution. Scanning Electron Microscopy (SEM) further confirmed the surface structure of the gel, showing irregular textures with bright areas that may represent aggregate formation, possibly enhancing the bioactive potential of the formulation. Differential Scanning Calorimetry (DSC) revealed an endothermic shift from 134.9°C in the pure extract to 135.6°C in the final formulation, suggesting increased thermal stability due to molecular interactions within the gel matrix-an essential factor for shelf-life and therapeutic performance. *In-vitro* drug release studies using dialysis bags demonstrated that 50% of the drug was released within 8 hours, followed by sustained release up to 90% over 168 hours (7 days). This controlled release pattern mimics the conditions within periodontal pockets, supporting prolonged drug availability (Figure 2 - see PDF). The *In vitro* antibacterial assessment showed increasing zones of inhibition against *Staphylococcus aureus* over time: 9.48 ± 1.97 mm on day 1, 18.49 ± 2.1 mm on day 3, 27.94 ± 1.46 mm on day 7 and 32.80 ± 2.4 mm on day 14 (Figure 3 - see PDF and Table 5 - see PDF). These results demonstrate that the *drynaria in-situ* gel possesses strong and sustained antibacterial activity, supporting its potential application in periodontal therapy.

## Discussion:

The progression of modern technology has produced better understanding of periodontal disease causation mechanisms together with disease development pathways. As a result development and subsequent acceptance of the use of multiple pharmacological agents. Periodontal disease exists as a pathogen that involves multiple germs. Downloads show that patients with chronic periodontitis yield better results when receiving scaling and root planing (SRP) along with adjuvant antibiotics rather than receiving SRP alone [14]. Surgery-resistant bacteria remain in position even after conventional SRP treatments fail to eliminate them from furcation areas and interproximal gaps and concavities and deep periodontal pockets because instruments cannot access such difficult-to-treat areas [15]. In such cases, bacteria may continue to exist in the root cementum and dentinal tubules even after comprehensive SRP, later migrating back to periodontal sites [5, 15]. Anti-infective therapies like topical, local, or systemic antibiotics can be used as adjuncts to mechanical periodontal treatment as a holistic approach towards management [14, 15]. Local drug delivery (LDD) systems are specifically valuable in this context, as they can deliver antibiotics directly to the target sites, maintain higher concentrations and offer long-term efficacy and good patient compliance [8]. The intended drug delivery system enhances periodontal treatment effects and provides patients with better compliance and satisfaction rates in addition to delivering efficient outcomes. Many medicinal plants and their constituents have been used in recent years to treat and prevent the progression of periodontal disease [16, 17]. Investigations have been conducted to evaluate *Drynaria quercifolia* phytochemistry and pharmacological applications *In vitro* and *In vivo* [18, 19 and 20]. Four ingredients are typically employed for extraction of *Drynaria rhizome*: petroleum ether, methanol, ethanol and chloroform, of which it has been observed that ethanol yielded the highest amount of, extract [21]. The dried ethanolic extract of *Drynaria rhizome* served as a base ingredient when creating our in-situ gel for achieving best possible results. Multiple animal researches have demonstrated that *Drynaria rhizome* possesses anti-inflammatory, analgesic and antipyretic properties [21, 22]. Research has evaluated *Drynaria quercifolia* plant (rhizome) extract as a bone-forming substitute for periodontal intraosseous defects treatment [11].

Ceasing the study focused on developing an evaluation of a *Drynaria quercifolia*-based gel usable for local drug delivery (LDD) applications to treat periodontal diseases while supplying a sustained natura alternative therapy for periodontitis management. As far as we know no investigation exists regarding the development of *Drynaria quercifolia* gel as a local drug delivery agent for periodontitis treatment. The research base consisted of acetic acid together with chitosan and poloxamer 407 and sodium tripolyphosphate (STPP). The natural deacetylation process produces chitosan which functions both as a permeabilizing and bioadhesive biopolymer material. Poloxamer gels (PG) serve as ideal thermosensitive polymers for local usage because their structure allows them to exist as solutions with low viscosity at room temperature and transform into gelphase at body temperature. Moreover, chitosan demonstrates non-toxic and biocompatible properties [23, 24 and 25]. The production of a body-temperature viscous gel required sodium tripolyphosphate (STPP) as a cross-linking agent [26, 27]. The consistent distribution of *Drynaria rhizome* extract within the gel was established by drug content evaluation because this ensures dependable treatment results. The gel exhibits temperature-sensitive characteristics because it adapts to the surrounding local conditions and maintains a steady drug release pattern which makes it suitable for periodontal therapy based on its viscosity profile measurements across different temperatures. The pH value of periodontal pockets measures 7.09 ± 0.07 without significant changes based on pocket depths [28]. The medication release mechanism of our tested drug-containing gel remained unaffected by local pH due to its mean pH measurement of 7.13. The consistency and stability of *Drynaria gel* formulation were confirmed through the analyses using Scanning electron microscopy (SEM), optical microscopy and FTIR spectroscopy tests. The prepared gel displayed thermal stability because of its results from Differential Scanning Calorimetry (DSC) thermogram analysis which ensures pharmaceutical stability and shelf life of this gel formulation. The formulation of a local drug delivery agent used in periodontal pockets must combine controlled drug release kinetics and pocket-retaining properties with easy manageability features [29]. The *Drynaria gel* revealed all essential characteristics which make it an effective LDD substance.

## Conclusion:

*Drynaria gel* has shown its capability to function as an efficient drug delivery method for treating periodontal diseases. The clinical assessment of the formulated gel as an auxiliary periodontal medication delivery requires clinical trials.
